# Age- and Sex-specific Prevalence, Longitudinal Trends and Factors of Cardiovascular-Kidney-Metabolic Syndrome Stages among Chinese Adults

**DOI:** 10.1038/s41514-026-00428-z

**Published:** 2026-06-16

**Authors:** Zhiyue Liang, Shiyuan Wei, Qi Gao, Shengli An, Licong Su

**Affiliations:** 1 Department of Biostatistics, School of Public Health (Guangdong Provincial Key Laboratory of Tropical Disease Research), Nanfang Hospital, Southern Medical University, Guangzhou, Guangdong China; 2 Department of Gynecology, The Affiliated Guangdong Second Provincial General Hospital of Jinan University, Guangzhou, China; 3 Department of Nephrology, Wuhan Fourth Hospital, Wuhan Clinical Research Center for Metabolic Chronic Kidney Disease, Wuhan, Hubei PR China; 4 Department of Biostatistics, School of Public Health (Guangdong Provincial Key Laboratory of Tropical Disease Research), Southern Medical University, Guangzhou, Guangdong China; 5 Division of Nephrology, National Clinical Research Center for Kidney and Urological Diseases, State Key Laboratory of Multi-organ Injury Prevention and Treatment, Guangdong Provincial Key Laboratory of Renal Failure Research, Guangdong Provincial Institute of Nephrology, Nanfang Hospital, Southern Medical University, Guangzhou, China

**Keywords:** Cardiology, Diseases, Health care, Medical research, Nephrology, Risk factors

## Abstract

Cardiovascular-Kidney-Metabolic (CKM) syndrome is uncharacterized in Chinese adults across ages, including centenarians. From three cohorts, age- and sex-standardized CKM prevalence showed nearly 90% stage≥1 and >18% advanced. Prevalence rose with age; advanced CKM reached 100% in male centenarians. Age, sex, smoking, and BMI were risk factors for advanced CKM, with faster male progression. These findings highlight growing disease burden in China’s aging population and inform future CKM prevention.

Cardio-kidney-metabolic (CKM) syndrome, introduced by the American Heart Association (AHA) in 2023^1^, is a clinical syndrome characterized by the interconnected pathophysiological interplay among cardiovascular disease (CVD), chronic kidney disease (CKD), and metabolic disorders. As reported, CKM syndrome has affected more than 25% of US adults between 2015 and 2020^2^, and was the leading causes of death in 2021^3^. In the US, nearly 90% of adults met criteria for at least stage 1 CKM syndrome from 2011 to 2020 and 15% met criteria for advanced stages^4^. Similarly, China faces a considerable burden from CKM syndrome, with the prevalence among adults rising from 77.1% in 2010 to 83.7% in 2019, affecting an estimated 875 million individuals^5^, particularly in middle-aged and older adults^6^.

However, few studies have compared disparities across different cohorts or examined CKM prevalence in centenarians. Using three nationally representative cohorts, we aimed to describe the age- and sex-standardized CKM syndrome prevalence, assess short-term longitudinal changes of CKM syndrome stages, explore the potential drivers and interaction factors of advanced CKM and clarify the major contributors of CKM stage 3.

The final study populations comprised 6604 adults from the China Health and Nutrition Survey (CHNS)^7^ 2009 wave (ages≥30), 9077 from the China Health and Retirement Longitudinal Study (CHARLS)^8^ 2011 wave (with 8,069 followed up in 2015, ages≥45), and 1,789 from the Chinese Longitudinal Healthy Longevity Survey (CLHLS)^9^ 2012 wave (with 1,035 followed up in 2014, ages≥65). Their mean (SD) ages were 53.5 (13.1), 59.5 (9.4), and 84.8 (11.9) years, with 53.6%, 53.5%, and 52.3% being female, respectively (Table S1–3). Overall, for CHNS in 2009, the age- and sex-standardized prevalence was 11.1% (95% CI, 10.2–11.9%) for stage 0, 12.7% (95% CI, 11.8%–13.5%) for stage 1, 58.2% (95% CI, 57.0–59.4%) for stage 2, 14.8% (95% CI, 14.2%–15.5%) for stage 3, and 3.2% (95% CI, 2.9–3.6%) for stage 4. For CHARLS in 2011, the standardized prevalence was 7.6% (95% CI, 7.1–8.2%) for stage 0, 14.2% (95% CI, 13.5–14.9%) for stage 1, 40.2% (95% CI, 39.2–41.1%) for stage 2, 24.8% (95% CI, 24.0–25.6%) for stage 3, and 13.2% (95% CI, 12.5%–13.9%) for stage 4. For CLHLS in 2012, the standardized prevalence was 3.7% (95% CI, 2.3–5.1%) for stage 0, 3.5% (95% CI, 2.0–4.9%) for stage 1, 27.0% (95% CI, 24.2–29.8%) for stage 2, 51.6% (95% CI, 48.9–54.3%) for stage 3, and 14.2% (95% CI, 11.8–16.7%) for stage 4 (Table 1). Crude prevalence estimates are provided in Table S4. During follow-up, the stage distribution changed significantly in CHARLS (overall *P* < 0.001), with stage 2 decreasing, stage 1 and 3 increasing (all *P* < 0.001); in CLHLS, the overall change was also significant (*P* = 0.002), driven by an increase in stage 0 and a decrease in stage 2 (Fig. 1).

**Fig. 1:**
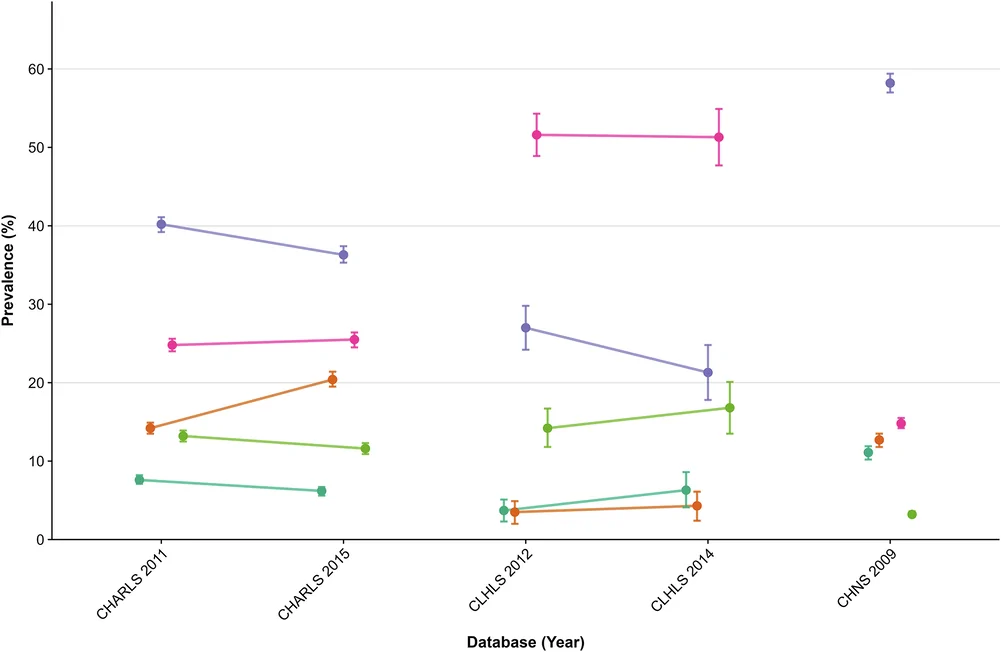
Prevalence and trend of CKM Stages Across Different Databases. The x-axis represents the different waves of the three cohorts, and the y-axis denotes the prevalence of different CKM stages among the population in a specific wave of each database. The prevalence rates for each time point are depicted as dots. The upper and lower extensions (error bars) represent the corresponding 95% confidence intervals. The segmented lines connecting the dots illustrate the changing trends of prevalence by CKM stages over the study period. The five stages (0-4) are color-coded as indicated in the legend. CHNS, China Health and Nutrition Survey; CHARLS, China Health and Retirement Longitudinal Study; CLHLS, Chinese Longitudinal Healthy Longevity; CKM, Cardiovascular-Kidney-Metabolic.

**Table 1:** Age- and Sex-Standardized Prevalence of Cardiovascular-Kidney-Metabolic Syndrome Stages Among Chinese Adults by Age and Sex in three national prospective cohorts

Cohorts	N	Adults 30 years or older by cardiovascular-kidney-metabolic syndrome stage				
		(Prevalence, % (95% CI))				
		Stage 0	Stage 1	Stage 2	Stage 3	Stage 4
**CHARLS**						
2011 Wave						
Total	9077	7.6 (7.1–8.2)	14.2 (13.5–14.9)	40.2 (39.2–41.1)	24.8 (24.0–25.6)	13.2 (12.5–13.9)
Age						
45–64 y	6544	9.0 (8.3–9.7)	17.0 (16.1–17.9)	45.3 (44.1–46.5)	17.4 (16.5–18.3)	11.3 (10.5–12.1)
≥ 65 y	2533	4.5 (3.5–5.4)	7.7 (6.5–8.9)	28.0 (26.1–30.0)	41.1 (39.0–43.1)	18.7 (17.0–20.5)
Sex						
Female	4856	6.2 (5.5–6.9)	16.4 (15.4–17.4)	52.8 (51.4–54.2)	9.5 (8.7–10.3)	15.1 (14.0–16.1)
Male	4221	9.1 (8.2–9.9)	12.0 (11.0–13.0)	27.6 (26.3–28.9)	40.0 (38.6–41.4)	11.4 (10.5–12.4)
2015 Wave						
Total	8069	6.2 (5.6–6.7)	20.4 (19.5–21.4)	36.3 (35.3–37.4)	25.5 (24.5–26.4)	11.6 (10.9–12.3)
Age						
45–64 y	4880	7.5 (6.8–8.3)	24.2 (23.0–25.4)	39.6 (38.3–40.9)	19.1 (18.0–20.2)	9.6 (8.8–10.4)
≥ 65 y	3189	3.3 (2.5–4.0)	13.1 (11.8–14.5)	29.1 (27.4–30.8)	37.7 (36.0–39.5)	16.8 (15.2–18.3)
Sex						
Female	4386	5.2 (4.5–5.8)	23.7 (22.4–25.0)	49.3 (47.8–50.8)	8.3 (7.6–9.1)	13.5 (12.5–14.6)
Male	3683	7.2 (6.3–8.1)	17.2 (15.9–18.6)	23.4 (21.9–24.9)	42.4 (40.8–44.1)	9.7 (8.7–10.7)
**CLHLS**						
2012 Wave						
Total	1789	3.7 (2.3–5.1)	3.5 (2.0–4.9)	27.0 (24.2–29.8)	51.6 (48.9–54.3)	14.2 (11.8–16.7)
Age						
<80 y	653	4.0 (2.3–5.7)	4.0 (2.3–5.7)	28.1 (24.7–31.5)	49.5 (46.4–52.7)	14.4 (11.5–17.3)
≥ 80 y	1136	2.3 (1.5–3.2)	1.1 (0.5–1.6)	21.7 (19.6–23.8)	61.3 (58.8–63.8)	13.6 (11.6–15.6)
Sex						
Female	936	5.1 (2.7–7.5)	5.4 (2.8–8.0)	49.6 (44.2–54.9)	23.5 (19.3–27.7)	16.4 (12.4–20.4)
Male	853	2.2 (0.9–3.4)	1.4 (0.4–2.4)	2.8 (1.3–4.2)	81.8 (78.6–85.0)	11.9 (9.3–14.5)
2014 Wave						
Total	1035	6.3 (4.1–8.6)	4.3 (2.4–6.1)	21.3 (17.8–24.8)	51.3 (47.7–54.9)	16.8 (13.5–20.1)
Age						
<80 y	423	6.7 (4.0–9.4)	4.6 (2.3–6.9)	21.6 (17.4–25.7)	49.6 (45.3–53.9)	17.5 (13.6–21.4)
≥ 80 y	612	4.4 (2.8–6.1)	2.6 (1.4–3.9)	20.1 (17.2–22.9)	59.3 (55.8–62.8)	13.6 (10.8–16.3)
Sex						
Female	494	9.4 (5.4–13.3)	6.5 (3.1–9.9)	38.3 (31.8–44.8)	26.9 (21.2–32.6)	18.9 (13.5–24.2)
Male	541	3.0 (1.2–4.9)	1.8 (0.4–3.3)	3.0 (1.2–4.9)	77.6 (73.3–81.8)	14.6 (11.0–18.1)
**CHNS**						
2009 Wave						
Total	6604	11.1 (10.2–11.9)	12.7 (11.8–13.5)	58.2 (57.0–59.4)	14.8 (14.2–15.5)	3.2 (2.9–3.6)
Age						
<45 y	1853	17.9 (16.2–19.6)	17.2 (15.5–18.9)	63.4 (61.3–65.6)	1.1 (0.6–1.6)	0.4 (0.1–0.7)
45–64 y	3346	7.3 (6.4–8.2)	11.2 (10.2–12.3)	61.6 (59.9–63.2)	16.7 (15.4–17.9)	3.2 (2.6–3.8)
≥ 65 y	1405	2.5 (1.5–3.5)	4.2 (3.0–5.5)	37.1 (34.4–39.8)	45.3 (42.4–48.1)	10.9 (8.9–12.9)
Sex						
Female	3540	13.0 (11.8–14.2)	16.0 (14.6–17.3)	61.9 (60.3–63.6)	6.0 (5.3–6.6)	3.2 (2.7–3.7)
Male	3,064	9.2 (8.1–10.4)	9.5 (8.3–10.6)	54.6 (52.8–56.3)	23.5 (22.3–24.6)	3.3 (2.7–3.9)

*CHNS* China Health and Nutrition Survey, *CHARLS* China Health and Retirement Longitudinal Study, *CLHLS* Chinese Longitudinal Healthy Longevity.

CKM prevalence increased progressively with age. In CHNS, adults aged ≥65 years had significantly higher prevalence of advanced stages (stages 3–4) than those aged 45–64 years and 30–44 years (56.2% vs. 19.9% vs. 1.5%, respectively; both *P* < 0.001; Table 1). This age‑related increase was consistently observed in both the CHARLS and CLHLS cohorts. Across all cohorts, the proportion of advanced CKM stages was significantly higher in men than in women (*P* < 0.001, Figure S1). Among CLHLS centenarians (2012 wave, *n* = 345), the prevalence by stage was: stage 0, 3.7% (95% CI, 1.8–5.6%); stage 1, 1.1% (0.0–2.1%); stage 2, 30.5% (26.2–34.8%); stage 3, 53.3% (48.6–58.0%); and stage 4, 11.4% (8.1–14.8%) (Table 2). Over 64.7% of centenarians had advanced CKM, reaching 100% in male centenarians (*n* = 61).

**Table 2:** Prevalence of cardiovascular-kidney-metabolic syndrome stages among Chinese adults aged 100 years old by sex in CLHLS

Cohorts	N	Adults 100 years or older by cardiovascular-kidney-metabolic syndrome stage (Prevalence, % (95% CI))				
		Stage 0	Stage 1	Stage 2	Stage 3	Stage 4
**CLHLS**						
2012 Wave						
Total	345	3.7 (1.8–5.6)	1.1 (0.0–2.1)	30.5 (26.2–34.8)	53.3 (48.6–58.0)	11.4 (8.1–14.8)
Sex						
Female	284	4.9 (2.4–7.4)	1.4 (0.0–2.8)	40.5 (34.8–46.2)	41.2 (35.5–46.9)	12.0 (8.2–15.7)
Male	61	0	0	0	90.2 (82.7–97.6)	9.8 (2.4–17.3)
2014 Wave						
Total	124	7.7 (3.2–14.2)	3.2 (0.1–6.0)	33.8 (26.4–41.3)	48.3 (41.1–55.5)	7.1 (2.6–11.6)
Sex						
Female	98	10.2 (4.2–16.2)	4.1 (0.2–8.0)	44.9 (35.1–54.7)	32.7 (23.4–41.9)	8.2 (2.7–13.6)
Male	26	0	0	0	96.2 (88.8–100.0)	3.8 (0.0–11.2)

*CLHLS* Chinese Longitudinal Healthy Longevity.

Multivariable logistic regression (adjusting for age, sex, marital status, education, smoking, drinking, and BMI) identified age, sex, smoking and BMI as consistent risk factors for advanced CKM (all *P* < 0.05, Table S5). Male sex was a strong risk factor in all cohort (CHARLS: OR = 2.22; CLHLS: OR = 23.22; CHNS: OR = 5.02). Age‑by‑sex interaction was significant in all cohorts (all *P* < 0.01, Table S6), indicating that the age‑related increase in advanced CKM is significantly steeper in males than in females. Decomposition of stage 3 into “CKD only”, “CVD risk only”, and “both” revealed that over 99% of Stage 3 cases were attributable to “CVD risk only” (e.g., CHARLS 2011: 99.3% in females, 99.1% in males), whereas “CKD only” or “both” accounted for less than 1% (Table S7). Thus, sex differences in Stage 3 are primarily driven by cardiovascular risk factors rather than CKD.

Sensitivity analysis for missing data showed that excluded participants had higher prevalence of diabetes and hypertension than those included (e.g. CHARLS 2011: 19.8% vs. 8.4% for diabetes; 51.7% vs. 41.2% for hypertension), with standardized mean differences (SMDs) of 0.333 and 0.211, respectively, suggesting that exclusion may lead to a slight underestimation of advanced CKM prevalence. Other variables showed good comparability (SMDs <0.20). Similar patterns were observed in CLHLS and CHNS, with SMDs for core metabolic conditions all <0.20, indicating low bias risk (Table S8–10). Thus, any bias from missing data is likely conservative.

Consistent with previous studies in US and other Chinese cohorts^4–6^, our study observed that the prevalence of CKM increases with age and exhibits significant sex-specific differences. Given the rapid aging of China’s population^10^, the disease burden of CKM is likely to escalate substantially. The standardized advanced CKM prevalence in CHNS 2009 (18.0%) is comparable to NHANES (15%)^4^. Males experience significantly faster age-related progression (per year: 7.8%, 5.7%, and 18.2% higher risk than females across CHARLS, CHNS, and CLHLS), in line with previous studies^11,12^ and suggesting men have greater cumulative cardiovascular risk exposure. Advanced CKM reached 100% in male centenarians. Possible mechanisms include higher smoking and alcohol use in male centenarians^13^, lack of estrogen’s cardio-renal protection^14,15^, and greater visceral adiposity^16^. Therefore, it is necessary to conduct earlier CKM screening and intervention for the male population in the future.

Multivariable logistic regression confirmed age, smoking, sex and BMI as robust risk factors for advanced CKM (all *P* < 0.05). Male sex had significantly higher odds of advanced CKM (CHARLS OR = 2.22, CLHLS OR = 23.22 and CHNS OR = 5.02) than female, which have consistently reported that these risk factors are significantly associated with CVD risk in previous study^17^. Early management of modifiable factors (smoking cessation, weight control) is crucial, especially in older males. The overwhelming dominance of CVD risk in stage 3 (>99%, Table S7) indicates that sex disparity in this stage is driven by cardiovascular risk factors rather than CKD, consistent with previous findings (very high-risk CKD accounts for only 2.5% in females and 3.2% in males)^18^. This may reflect the high incidence and mortality of CVD^19^. Therefore, controlling cardiovascular risk may be more effective than focusing on CKD in preventing advanced CKM.

Given the high disease burden of CKM syndrome, we proposed a tiered, integrated management strategy for it, including prevention, treatment, and rehabilitation. First, as the “last kilometer” close to residents in both urban and rural areas, community/township health service centers serve as the peripheral nodes of the basic public health service network and function as the primary institutions for CKM screening, management of stage 1 CKM and health education. Second, county/city secondary hospitals serve as the primary institutions for standardized treatment of moderate-to-advanced CKM and management of complications. Third, provincial/city tertiary hospitals are responsible for the treatment of advanced CKM patients with complex and critical condition and for providing treatment guidance to lower-level hospitals (for example, using teleconsultation technology). Fourth, patients after receiving treatment at higher-level hospitals will return to community/township health service centers or rehabilitation hospitals for subsequent rehabilitative care. Our strategy will integrate medical institutions within the region, share resources, and enable smoother tiered care.

In addition, among men aged over 65 years, especially centenarians, the prevalence of advanced‑stage CKM is extremely high. Enhanced regular CVD risk assessment, CKD screening and follow up, and clinical medication management should be emphasized in this population. For these special groups, current healthcare management policies must be further improved. First, we suggest that national health insurance should increase the reimbursement rate and expand coverage for this population, thereby encouraging them to attend follow‑up visits for treatment evaluation and improving adherence. Second, community physicians should strengthen regular follow‑up for this population, either online or interview.

One strength of our study is the use of three nationally representative cohorts to systematically elucidate the prevalence of CKM syndrome across diverse populations. Age and sex standardization enabled meaningful comparability across three national cohorts with inherently different age structures. Furthermore, the key cardiovascular risk factors and the age‑by‑sex interaction were highly consistent across all cohorts, confirming the reproducibility of the findings. However, limitations include potential selection bias due to missing data from some participants, the reliance on self-reported CVD definitions, finite follow-up due to blood test availability and small male centenarian sample size (*n* = 61 in 2012 wave and *n* = 26 in 2014 wave), warranting cautious interpretation.

In conclusion, this study underscores the unprecedented challenge of CKM control posed by China’s intensifying population aging. To address this, concerted efforts by health authorities are urgently needed, focusing on strengthening weight management, improving lifestyle habits, and universalizing regular health examinations.

## Methods

### Study Population

We utilized three nationally representative longitudinal databases covering progressively broader age ranges, including the CHNS (2009 wave)^7^, CHARLS (2011–2015 wave)^8^, and CLHLS (2012–2014 wave)^9^, respectively. These cohorts included nationally representative sample of the Chinese population using multistage probability design or targeted sampling strategy and incorporated interviews, physical examinations, and laboratory measurements. Response rates for CHARLS and CLHLS were 87% and 88%, respectively. Adults aged 30 years or older who underwent physical examination and fasting laboratory testing were included. We excluded participants with missing data required for CKM staging.

### Definition of CKM Syndrome

For each participant, we determined the CKM syndrome stage^1^: stage 0 (no CKM risk factors), 1 (excess or dysfunctional adiposity), 2 [additional metabolic risk factors or moderate- or high-risk chronic kidney disease (CKD)], 3 (very high-risk CKD or high predicted 10-year CVD risk), or 4 (established CVD). Detailed descriptions of stage definitions, are shown in the Table S11. Advanced CKM syndrome stages were defined as stages 3 or 4.

### Statistical Analysis

Continuous variables with normal distribution are presented as mean (SD), and those with non‑normal distribution as median (IQR); categorical variables are presented as n (%). Group comparisons were performed using ANOVA, Kruskal-Wallis test, or χ² test, as appropriate. Prevalence is presented as percentage with 95% confidence intervals (CIs). Age‑ and sex‑standardized prevalence was calculated using the direct method with the 2010 Chinese census as the standard population. Multivariable logistic regression was used to identify factors associated with advanced CKM (stages 3–4), adjusting for age, sex, marital status, education, smoking, drinking, and BMI; age‑by‑sex interaction was tested in each cohort. Longitudinal trends in CHARLS and CLHLS were assessed using overall chi‑square tests and stage‑specific proportion comparisons. Sensitivity analysis for missing data was performed by comparing baseline characteristics of included versus excluded participants. All analyses were conducted using R version 4.3.3, and a two‑sided *P* < 0.05 was considered statistically significant.

The Institutional Review Board of Nanfang hospital waived this study because these databases are deidentified and publicly used.

## Supplementary information

Supplementary Material

## Data Availability

The data supporting this study are publicly available on the China Health and Nutrition Survey repository (http://www.cpc.unc.edu/projects/china), China Health and Retirement Longitudinal Study repository (https://charls.pku.edu.cn/), and Chinese Longitudinal Healthy Longevity Survey repository (https://opendata.pku.edu.cn/).

## References

[1] Ndumele, C. E. et al. A synopsis of the evidence for the science and clinical management of cardiovascular-kidney-metabolic (ckm) syndrome: a scientific statement from the american heart association. *Circulation.* **148**, 1636–1664 (2023). 10.1161/CIR.0000000000001186 37807920

[2] Ostrominski, J. W. et al. Prevalence and overlap of cardiac, renal, and metabolic conditions in us adults, 1999-2020. *JAMA Cardiol.* **8**, 1050–1060 (2023). 10.1001/jamacardio.2023.3241 PMC10535010 37755728

[3] Xu, J., Murphy, S. L., Kochanek, K. D. & Arias, E. Mortality in the United States, 2021. *NCHS Data Brief.* **456**, 1–8 (2022). 36598387

[4] Aggarwal, R., Ostrominski, J. W. & Vaduganathan, M. Prevalence of cardiovascular-kidney-metabolic syndrome stages in us adults, 2011-2020. *JAMA* **331**, 1858–1860 (2024). 10.1001/jama.2024.6892 PMC11079779 38717747

[5] He, D. et al. Rising prevalence of cardiovascular-kidney-metabolic syndrome in china, 2010-2019 national cross-sectional surveys. *J. Am. Coll. Cardiol.* **86**, 213–216 (2025). 10.1016/j.jacc.2025.05.030 40669959

[6] Zhang, H. et al. Prevalence of cardiovascular-kidney-metabolic syndrome stages among middle-aged and older adults in china. *JACC Asia.* **5**, 393–395 (2025). 10.1016/j.jacasi.2024.12.003 PMC11934037 40049933

[7] Popkin, B. M., Du, S., Zhai, F. & Zhang, B. Cohort profile: the China Health and Nutrition Survey-monitoring and understanding socio-economic and health change in China, 1989-2011. *Int. J. Epidemiol.* **39**, 1435–1440 (2010). 10.1093/ije/dyp322 PMC2992625 19887509

[8] Zhao, Y., Hu, Y., Smith, J. P., Strauss, J. & Yang, G. Cohort profile: the China Health and Retirement Longitudinal Study (CHARLS). *Int. J. Epidemiol.* **43**, 61–68 (2014). 10.1093/ije/dys203 PMC3937970 23243115

[9] Gu, D., Dupre, M. E., Warner, D. F. & Zeng, Y. Changing health status and health expectancies among older adults in China: gender differences from 1992 to 2002. *Soc. Sci. Med.* **68**, 2170–2179 (2009). 10.1016/j.socscimed.2009.03.031 PMC2727476 19394120

[10] Gruber, J., Lin, M., Yang, H. & Yi, J. China’s social health insurance in the era of rapid population aging. *JAMA Health Forum.* **4**, e251105 (2025). 10.1001/jamahealthforum.2025.1105 40279110

[11] Pana, T. A. et al. Sex-specific lifetime risk of cardiovascular events: the European Prospective Investigation into Cancer-Norfolk prospective population cohort study. *Eur. J. Prev. Cardiol.* **1**, 230–241 (2024). 10.1093/eurjpc/zwad283 PMC10809170 38031203

[12] Bugeja, A. et al. Sex-related disparities in cardiovascular outcomes among older adults with late-onset hypertension. *Hypertension.* **81**, 1583–1591 (2024). 10.1161/HYPERTENSIONAHA.124.22870 PMC11177607 38660798

[13] Mukamal, K. J. The effects of smoking and drinking on cardiovascular disease and risk factors. *Alcohol Res. Health.* **29**, 199–202 (2006). PMC6527044 17373409

[14] Valdivielso, J. M., Jacobs-Cachá, C. & Soler, M. J. Sex hormones and their influence on chronic kidney disease. *Curr. Opin. Nephrol. Hypertens.* **28**, 1–9 (2019). 10.1097/MNH.0000000000000463 30320621

[15] Visniauskas, B. et al. Estrogen-mediated mechanisms in hypertension and other cardiovascular diseases. *J. Hum. Hypertens.* **37**, 609–618 (2023). 10.1038/s41371-022-00771-0 PMC10919324 36319856

[16] Shetty S., Suvarna R., Bhattacharya S., Seetharaman K. Visceral adiposity and cardiometabolic risk: clinical insights and assessment. *Cardiol. Rev*. (2025). 10.1097/CRD.0000000000000984 40622164

[17] Global Cardiovascular Risk Consortium et al. Global effect of modifiable risk factors on cardiovascular disease and mortality. *N. Engl. J. Med.* **389**, 1273–1285 (2023). 10.1056/NEJMoa2206916 PMC10589462 37632466

[18] Liu, L. in, Cai, A. nping, Cheng, Y. unjiu & Cheung, B. ernardM. Y. Sex differences in cardiovascular-kidney-metabolic syndrome and their first adverse cardiovascular outcomes. *J. Hypertension.* **43**, e28 (2025).

[19] Mensah, G. et al. Comparable estimates of mortality and trends for cardiovascular diseases including congenital heart disease in 21 world regions in 1990 and 2010: the global burden of diseases, injuries and risk factors study. *JACC* **61**, E1406 (2013).

