# Supplementary material for: Age- and Sex-specific Prevalence, Longitudinal Trends and Factors of Cardiovascular-Kidney-Metabolic Syndrome Stages among Chinese Adults
Source: NPJ Aging. 2026 Jun 16;12(1):136. doi: 10.1038/s41514-026-00428-z (PMC13635093; doi:10.1038/s41514-026-00428-z)
Supplement: Supplementary file 1 [file 41514_2026_428_MOESM1_ESM.docx]

**Supplementary Material Content**

**Supplementary Tables**

**Table S1.** Baseline characteristics of the CHARLS 2011 cohort by CKM stage.

**Table S2.** Baseline characteristics of the CLHLS 2012 cohort by CKM stage.

**Table S3.** Baseline characteristics of the CHNS 2009 cohort by CKM stage.

**Table S4.** Crude prevalence of Cardiovascular-Kidney-Metabolic Syndrome Stages Among Chinese Adults by Age and Sex in Three National Prospective Cohorts.

**Table S5.** Multivariable logistic regression for advanced CKM in three cohorts.

**Table S6.** Age-by-sex interaction for advanced CKM in three cohorts.

**Table S7.** Decomposition of Stage 3 CKM: contributions of CKD and CVD risk.

**Table S8.** Comparison of baseline characteristics between included and excluded participants in the CHARLS 2011 cohort.

**Table S9.** Comparison of baseline characteristics between included and excluded participants in the CLHLS 2012 cohort.

**Table S10.** Comparison of baseline characteristics between included and excluded participants in the CHNS 2009 cohort.

**Table S11.** Definition of CKM syndrome staging.

**Supplementary Figure**

**Figure S1.** Prevalence and trends by gender in cardio-kidney-metabolic syndrome stages 1-2 vs. 3-4.

**STROBE Statement**—checklist of items that should be included in reports of observational studies

**Table S1. Baseline characteristics of the CHARLS 2011 cohort by CKM stage.**

| Variables | Overall | CKM Stage | | | | | P |
| --- | --- | --- | --- | --- | --- | --- | --- |
|  |  | 0 | 1 | 2 | 3 | 4 |  |
| n | 9077 | 677 | 1302 | 3706 | 2169 | 1223 |  |
| Age, years (mean (SD)) | 59.53 (9.38) | 56.63 (7.96) | 55.68 (7.81) | 56.92 (8.47) | 65.64 (8.78) | 62.29 (9.18) | <0.001 |
| Sex (%) |  |  |  |  |  |  | <0.001 |
| Female | 4856 (53.50) | 307 (45.35) | 815 (62.60) | 2591 (69.91) | 415 (19.13) | 728 (59.53) |  |
| Male | 4221 (46.50) | 370 (54.65) | 487 (37.40) | 1115 (30.09) | 1754 (80.87) | 495 (40.47) |  |
| Marriage (%) |  |  |  |  |  |  | <0.001 |
| Married | 7972 (87.83) | 620 (91.58) | 1187 (91.17) | 3318 (89.53) | 1830 (84.37) | 1017 (83.16) |  |
| Not married | 1105 (12.17) | 57 (8.42) | 115 (8.83) | 388 (10.47) | 339 (15.63) | 206 (16.84) |  |
| Education (%) |  |  |  |  |  |  | <0.001 |
| >=primary high school | 2652 (29.22) | 202 (29.84) | 417 (32.03) | 1162 (31.35) | 530 (24.45) | 341 (27.88) |  |
| Illiterate | 2666 (29.37) | 182 (26.88) | 374 (28.73) | 1172 (31.62) | 591 (27.26) | 347 (28.37) |  |
| Primary school | 3758 (41.41) | 293 (43.28) | 511 (39.25) | 1372 (37.02) | 1047 (48.29) | 535 (43.74) |  |
| Smoke (%) |  |  |  |  |  |  | <0.001 |
| Ex-smoker | 797 (8.78) | 52 (7.68) | 85 (6.53) | 284 (7.66) | 217 (10.00) | 159 (13.00) |  |
| Non-smoker | 5527 (60.89) | 384 (56.72) | 910 (69.89) | 2884 (77.82) | 578 (26.65) | 771 (63.04) |  |
| Smoker | 2753 (30.33) | 241 (35.60) | 307 (23.58) | 538 (14.52) | 1374 (63.35) | 293 (23.96) |  |
| Drink (%) |  |  |  |  |  |  | <0.001 |
| No | 6099 (67.19) | 418 (61.74) | 894 (68.66) | 2712 (73.18) | 1147 (52.88) | 928 (75.88) |  |
| Yes | 2978 (32.81) | 259 (38.26) | 408 (31.34) | 994 (26.82) | 1022 (47.12) | 295 (24.12) |  |
| Height, cm (mean (SD)) | 157.82 (9.07) | 158.48 (8.74) | 157.10 (8.30) | 156.55 (8.84) | 160.31 (9.45) | 157.68 (9.12) | <0.001 |
| Weight, Kg (mean (SD)) | 58.81 (11.63) | 50.68 (7.26) | 57.25 (10.62) | 59.49 (11.45) | 59.95 (11.98) | 60.92 (12.47) | <0.001 |
| BMI (mean (SD)) | 24.00 (27.71) | 20.11 (1.71) | 23.12 (3.47) | 24.41 (10.72) | 23.71 (16.71) | 26.36 (69.48) | <0.001 |
| Creatinine, mg/dL (median [IQR]) | 0.76 [0.66, 0.88] | 0.75 [0.63, 0.85] | 0.71 [0.62, 0.81] | 0.72 [0.63, 0.85] | 0.85 [0.73, 0.97] | 0.77 [0.66, 0.90] | <0.001 |
| Glucose, mg/dL (median [IQR]) | 102.60 [94.50, 113.76] | 92.52 [87.48, 96.66] | 101.52 [94.18, 107.46] | 103.32 [95.58, 113.36] | 106.92 [96.66, 127.62] | 103.86 [95.94, 117.09] | <0.001 |
| HbA1c, % (median [IQR]) | 5.10 [4.90, 5.40] | 5.00 [4.80, 5.20] | 5.10 [4.90, 5.40] | 5.10 [4.90, 5.40] | 5.20 [4.90, 5.60] | 5.20 [4.90, 5.50] | <0.001 |
| HDL, mg/dL (median [IQR]) | 49.48 [40.59, 59.92] | 56.06 [48.33, 65.34] | 56.83 [49.10, 66.40] | 48.33 [39.82, 58.38] | 44.85 [37.50, 55.67] | 46.78 [38.27, 57.99] | <0.001 |
| LDL, mg/dL (median [IQR]) | 114.43 [93.17, 137.63] | 105.16 [88.14, 125.64] | 114.82 [95.10, 135.21] | 114.05 [93.17, 136.86] | 117.14 [94.33, 142.27] | 116.37 [94.33, 138.98] | <0.001 |
| TC, mg/dL (median [IQR]) | 190.59 [167.40, 215.34] | 176.29 [157.35, 199.49] | 186.73 [165.46, 210.60] | 190.98 [168.56, 216.11] | 195.62 [170.10, 222.29] | 191.75 [168.36, 216.88] | <0.001 |
| TG, mg/dL (median [IQR]) | 105.32 [75.22, 153.99] | 76.11 [59.29, 96.46] | 80.54 [63.72, 101.78] | 124.79 [84.07, 173.46] | 114.17 [79.65, 174.35] | 115.93 [80.54, 170.36] | <0.001 |
| eGFR, ml/min/1.72m^2^ (median [IQR]) | 86.00 [70.68, 98.03] | 89.71 [75.43, 99.96] | 93.73 [80.42, 101.95] | 90.98 [76.71, 100.72] | 72.33 [60.77, 85.86] | 83.63 [67.01, 95.32] | <0.001 |
| SBP, mmHg (mean (SD)) | 130.66 (21.51) | 110.92 (9.35) | 113.55 (8.72) | 131.31 (19.19) | 143.74 (21.77) | 134.64 (22.37) | <0.001 |
| DBP, mmHg (mean (SD)) | 75.82 (12.15) | 65.61 (7.39) | 67.38 (7.03) | 77.73 (11.77) | 80.02 (12.09) | 77.21 (12.49) | <0.001 |
| Diabetes (%) |  |  |  |  |  |  | <0.001 |
| No | 8318 (91.64) | 677 (100.00) | 1302 (100.00) | 3431 (92.58) | 1878 (86.58) | 1030 (84.22) |  |
| Yes | 759 (8.36) | 0 (0.00) | 0 (0.00) | 275 (7.42) | 291 (13.42) | 193 (15.78) |  |
| Hypertension (%) |  |  |  |  |  |  | <0.001 |
| No | 5334 (58.76) | 677 (100.00) | 1302 (100.00) | 2047 (55.23) | 821 (37.85) | 487 (39.82) |  |
| Yes | 3743 (41.24) | 0 (0.00) | 0 (0.00) | 1659 (44.77) | 1348 (62.15) | 736 (60.18) |  |
| Dyslipidemia (%) |  |  |  |  |  |  | <0.001 |
| No | 6839 (75.34) | 637 (94.09) | 1159 (89.02) | 2699 (72.83) | 1473 (67.91) | 871 (71.22) |  |
| Yes | 2238 (24.66) | 40 (5.91) | 143 (10.98) | 1007 (27.17) | 696 (32.09) | 352 (28.78) |  |

**Abbreviations**: CHARLS, China Health and Retirement Longitudinal Study; CKM syndrome, Cardiovascular-Kidney-Metabolic syndrome; BMI, Body Mass Index; HbA1c, Hemoglobin A1c; HDL, High-Density Lipoprotein; LDL, Low-Density Lipoprotein; TC, Total Cholesterol; TG, Triglycerides; eGFR, estimated Glomerular Filtration Rate; SBP, Systolic Blood Pressure; DBP, Diastolic Blood Pressure.

**Table S2. Baseline characteristics of the CLHLS 2012 cohort by CKM stage.**

| Variables | Overall | CKM Stage | | | | | P |
| --- | --- | --- | --- | --- | --- | --- | --- |
|  |  | 0 | 1 | 2 | 3 | 4 |  |
| n | 1789 | 52 | 34 | 399 | 1061 | 243 |  |
| Age, years (mean (SD)) | 84.78 (11.91) | 83.77 (13.98) | 78.29 (12.44) | 86.89 (12.86) | 84.40 (11.47) | 84.15 (11.07) | <0.001 |
| Sex (%) |  |  |  |  |  |  | <0.001 |
| Female | 936 (52.32) | 41 (78.85) | 27 (79.41) | 385 (96.49) | 346 (32.61) | 137 (56.38) |  |
| Male | 853 (47.68) | 11 (21.15) | 7 (20.59) | 14 (3.51) | 715 (67.39) | 106 (43.62) |  |
| Marriage (%) |  |  |  |  |  |  | <0.001 |
| Married | 756 (42.26) | 23 (44.23) | 19 (55.88) | 110 (27.57) | 511 (48.16) | 93 (38.27) |  |
| Not married | 1033 (57.74) | 29 (55.77) | 15 (44.12) | 289 (72.43) | 550 (51.84) | 150 (61.73) |  |
| Education (%) |  |  |  |  |  |  | <0.001 |
| >=primary high school | 179 (10.01) | 4 (7.69) | 5 (14.71) | 21 (5.26) | 126 (11.88) | 23 (9.47) |  |
| Illiterate | 1096 (61.26) | 38 (73.08) | 22 (64.71) | 324 (81.20) | 561 (52.87) | 151 (62.14) |  |
| Primary school | 514 (28.73) | 10 (19.23) | 7 (20.59) | 54 (13.53) | 374 (35.25) | 69 (28.40) |  |
| Smoke (%) |  |  |  |  |  |  | <0.001 |
| Ex-smoker | 159 (8.89) | 3 (5.77) | 3 (8.82) | 16 (4.01) | 108 (10.18) | 29 (11.93) |  |
| Non-smoker | 1311 (73.28) | 46 (88.46) | 29 (85.28) | 371 (92.98) | 675 (63.62) | 190 (78.19) |  |
| Smoker | 319 (17.83) | 3 (5.77) | 2 (5.88) | 12 (3.01) | 278 (26.20) | 24 (9.88) |  |
| Drink (%) |  |  |  |  |  |  | <0.001 |
| No | 1495 (83.57) | 46 (88.46) | 29 (85.29) | 372 (93.23) | 829 (78.13) | 219 (90.12) |  |
| Yes | 294 (16.43) | 6 (11.54) | 5 (14.71) | 27 (6.77) | 232 (21.87) | 24 (9.88) |  |
| Height, cm (mean (SD)) | 154.20 (11.05) | 147.54 (10.15) | 150.32 (12.59) | 148.46 (9.03) | 156.65 (11.01) | 154.86 (10.07) | <0.001 |
| Weight, Kg (mean (SD)) | 51.15 (13.20) | 41.85 (6.79) | 55.41 (20.43) | 45.51 (10.40) | 53.10 (13.47) | 53.29 (12.39) | <0.001 |
| BMI (mean (SD)) | 21.35 (4.32) | 19.18 (1.99) | 24.36 (8.63) | 20.56 (3.80) | 21.50 (4.37) | 22.05 (3.91) | <0.001 |
| Creatinine, mg/dL (median [IQR]) | 0.87 [0.72, 1.03] | 0.69 [0.62, 0.79] | 0.72 [0.64, 0.77] | 0.81 [0.68, 0.95] | 0.92 [0.77, 1.07] | 0.87 [0.72, 1.03] | <0.001 |
| Glucose, mg/dL (median [IQR]) | 4.48 [3.76, 5.18] | 4.19 [3.59, 4.80] | 4.30 [3.71, 4.98] | 4.43 [3.74, 5.02] | 4.51 [3.74, 5.26] | 4.59 [3.96, 5.25] | 0.035 |
| HDL, mmol/l (median [IQR]) | 1.24 [1.03, 1.49] | 1.23 [1.05, 1.45] | 1.25 [1.01, 1.48] | 1.25 [1.02, 1.50] | 1.24 [1.04, 1.49] | 1.23 [1.02, 1.48] | 0.993 |
| LDL, mmol/l (median [IQR]) | 2.51 [1.99, 3.05] | 2.57 [2.03, 2.92] | 2.43 [1.99, 3.22] | 2.64 [2.03, 3.18] | 2.47 [1.98, 3.00] | 2.48 [1.99, 3.06] | 0.129 |
| TC, mmol/l (median [IQR]) | 4.24 [3.61, 4.92] | 4.19 [3.64, 4.80] | 4.22 [3.43, 4.90] | 4.43 [3.71, 5.13] | 4.20 [3.58, 4.81] | 4.21 [3.70, 4.88] | 0.025 |
| TG, mmol/l (median [IQR]) | 0.81 [0.59, 1.16] | 0.64 [0.52, 0.94] | 0.78 [0.56, 1.00] | 0.85 [0.64, 1.22] | 0.80 [0.59, 1.14] | 0.85 [0.60, 1.23] | 0.002 |
| eGFR, ml/min/1.72m^2^ (median [IQR]) | 61.20 [48.82, 74.51] | 76.10 [70.04, 81.57] | 76.87 [71.56, 86.67] | 65.77 [52.70, 77.88] | 58.57 [46.77, 71.56] | 62.06 [49.62, 74.15] | <0.001 |
| SBP, mmHg (mean (SD)) | 140.19 (22.76) | 111.55 (10.97) | 114.84 (9.38) | 131.34 (12.92) | 145.50 (23.67) | 141.23 (23.22) | <0.001 |
| DBP, mmHg (mean (SD)) | 80.87 (12.00) | 69.13 (6.91) | 70.31 (6.21) | 78.98 (10.23) | 82.18 (12.15) | 82.23 (13.02) | <0.001 |
| Diabetes (%) |  |  |  |  |  |  | <0.001 |
| No | 1633 (91.28) | 52 (100.00) | 34 (100.00) | 394 (98.75) | 943 (88.88) | 210 (86.42) |  |
| Yes | 156 (8.72) | 0 (0.00) | 0 (0.00) | 5 (1.25) | 118 (11.12) | 33 (13.58) |  |
| Hypertension (%) |  |  |  |  |  |  | <0.001 |
| No | 717 (40.08) | 52 (100.00) | 34 (100.00) | 208 (52.13) | 346 (32.61) | 77 (31.69) |  |
| Yes | 1072 (59.92) | 0 (0.00) | 0 (0.00) | 191 (47.87) | 715 (67.39) | 166 (68.31) |  |
| Dyslipidemia (%) |  |  |  |  |  |  | <0.001 |
| No | 1368 (76.47) | 48 (92.31) | 29 (85.29) | 280 (70.18) | 835 (78.70) | 176 (72.43) |  |
| Yes | 421 (23.53) | 4 (7.69) | 5 (14.71) | 119 (29.82) | 226 (21.30) | 67 (27.57) |  |

**Abbreviations**: CLHLS, Chinese Longitudinal Healthy Longevity; CKM syndrome, Cardiovascular-Kidney-Metabolic syndrome; BMI, Body Mass Index; HbA1c, Hemoglobin A1c; HDL, High-Density Lipoprotein; LDL, Low-Density Lipoprotein; TC, Total Cholesterol; TG, Triglycerides; eGFR, estimated Glomerular Filtration Rate; SBP, Systolic Blood Pressure; DBP, Diastolic Blood Pressure.

**Table S3. Baseline characteristics of the CHNS 2009 cohort by CKM stage.**

| Variables | Overall | CKM Stage | | | | | P |
| --- | --- | --- | --- | --- | --- | --- | --- |
|  |  | 0 | 1 | 2 | 3 | 4 |  |
| n | 6604 | 613 | 764 | 3741 | 1212 | 274 |  |
| Age, years (mean (SD)) | 53.51 (13.05) | 44.71 (10.60) | 47.73 (10.74) | 51.24 (11.64) | 65.82 (9.72) | 65.93 (10.26) | <0.001 |
| Sex (%) |  |  |  |  |  |  | <0.001 |
| Female | 3540 (53.60) | 372 (60.69) | 498 (65.18) | 2262 (60.47) | 265 (21.86) | 143 (52.19) |  |
| Male | 3064 (46.40) | 241 (39.31) | 266 (34.82) | 1479 (39.53) | 947 (78.14) | 131 (47.81) |  |
| Marriage (%) |  |  |  |  |  |  | <0.001 |
| Married | 5809 (87.96) | 563 (91.84) | 716 (93.72) | 3304 (88.32) | 998 (82.34) | 228 (83.21) |  |
| Not married | 795 (12.04) | 50 (8.16) | 48 (6.28) | 437 (11.68) | 214 (17.66) | 46 (16.79) |  |
| Education (%) |  |  |  |  |  |  | <0.001 |
| >=primary high school | 1384 (20.96) | 154 (25.12) | 157 (20.55) | 809 (21.63) | 205 (16.91) | 59 (21.53) |  |
| Illiterate | 1758 (26.62) | 98 (15.99) | 167 (21.86) | 970 (25.93) | 426 (35.15) | 97 (35.40) |  |
| Primary school | 3462 (52.42) | 361 (58.89) | 440 (57.59) | 1962 (52.45) | 581 (47.94) | 118 (43.07) |  |
| Smoke (%) |  |  |  |  |  |  | <0.001 |
| Ex-smoker | 223 (3.38) | 11 (1.79) | 15 (1.96) | 93 (2.49) | 76 (6.27) | 28 (10.22) |  |
| Non-smoker | 4521 (68.46) | 433 (70.64) | 591 (77.36) | 2846 (76.16) | 457 (37.71) | 191 (69.71) |  |
| Smoker | 1860 (28.16) | 169 (27.57) | 158 (20.68) | 799 (21.36) | 679 (56.02) | 55 (20.07) |  |
| Drink (%) |  |  |  |  |  |  | <0.001 |
| No | 4432 (67.11) | 416 (67.86) | 543 (71.07) | 2578 (68.91) | 674 (55.61) | 221 (80.66) |  |
| Yes | 2172 (32.89) | 197 (32.14) | 221 (28.93) | 1163 (31.09) | 538 (44.39) | 53 (19.34) |  |
| Height, cm (mean (SD)) | 160.37 (8.48) | 159.61 (7.83) | 159.45 (8.00) | 160.08 (8.70) | 162.34 (8.14) | 159.89 (8.34) | <0.001 |
| Weight, Kg (mean (SD)) | 60.78 (11.22) | 51.65 (6.59) | 58.88 (8.97) | 61.67 (11.23) | 63.36 (11.98) | 63.03 (10.81) | <0.001 |
| BMI (mean (SD)) | 23.55 (3.45) | 20.22 (1.62) | 23.12 (2.78) | 23.97 (3.37) | 23.96 (3.72) | 24.63 (3.67) | <0.001 |
| Creatinine, mg/dL (median [IQR]) | 0.96 [0.86, 1.10] | 0.93 [0.84, 1.04] | 0.90 [0.83, 1.01] | 0.95 [0.85, 1.07] | 1.07 [0.96, 1.22] | 1.02 [0.89, 1.21] | <0.001 |
| Glucose, mg/dL (median [IQR]) | 93.24 [85.86, 102.60] | 86.58 [81.54, 91.62] | 91.04 [84.60, 99.59] | 93.47 [86.40, 101.90] | 99.95 [90.00, 120.84] | 98.19 [89.42, 112.10] | <0.001 |
| HbA1c, % (median [IQR]) | 5.50 [5.20, 5.90] | 5.20 [5.00, 5.40] | 5.50 [5.20, 5.80] | 5.50 [5.20, 5.90] | 5.80 [5.40, 6.40] | 5.80 [5.50, 6.20] | <0.001 |
| HDL, mg/dL (median [IQR]) | 53.75 [45.24, 63.42] | 59.55 [51.04, 68.06] | 57.23 [50.27, 66.13] | 53.36 [44.86, 62.65] | 49.50 [41.76, 61.48] | 49.88 [41.09, 59.16] | <0.001 |
| LDL, mg/dL (median [IQR]) | 115.24 [93.19, 139.21] | 101.31 [83.91, 121.42] | 112.34 [93.10, 133.89] | 115.62 [93.97, 140.37] | 121.81 [98.99, 147.33] | 121.62 [96.96, 143.08] | <0.001 |
| TC, mg/dL (median [IQR]) | 187.55 [164.35, 213.84] | 168.99 [147.72, 191.42] | 178.27 [157.00, 201.08] | 188.71 [165.89, 216.16] | 198.96 [174.01, 225.44] | 192.19 [166.76, 218.10] | <0.001 |
| TG, mg/dL (median [IQR]) | 114.26 [77.95, 176.26] | 76.17 [57.57, 98.32] | 80.60 [61.12, 103.63] | 133.75 [86.80, 193.09] | 135.52 [91.23, 221.43] | 138.62 [92.12, 205.93] | <0.001 |
| eGFR, ml/min/1.72m^2^ (median [IQR]) | 76.78 [66.45, 87.22] | 82.21 [73.93, 91.52] | 81.59 [73.92, 91.51] | 77.88 [67.88, 87.94] | 67.70 [56.64, 78.55] | 65.41 [55.02, 76.70] | <0.001 |
| SBP, mmHg (mean (SD)) | 126.34 (19.21) | 108.15 (9.58) | 111.57 (9.29) | 125.89 (16.11) | 142.80 (19.36) | 141.52 (21.62) | <0.001 |
| DBP, mmHg (mean (SD)) | 80.93 (11.18) | 69.71 (6.02) | 71.49 (5.68) | 82.49 (9.83) | 86.75 (12.03) | 85.16 (12.39) | <0.001 |
| Diabetes (%) |  |  |  |  |  |  | <0.001 |
| No | 6384 (96.67) | 613 (100.00) | 764 (100.00) | 3679 (98.34) | 1086 (89.60) | 242 (88.32) |  |
| Yes | 220 (3.33) | 0 (0.00) | 0 (0.00) | 62 (1.66) | 126 (10.40) | 32 (11.68) |  |
| Hypertension (%) |  |  |  |  |  |  | <0.001 |
| No | 5626 (85.19) | 613 (100.00) | 764 (100.00) | 3348 (89.49) | 785 (64.77) | 116 (42.34) |  |
| Yes | 978 (14.81) | 0 (0.00) | 0 (0.00) | 393 (10.51) | 427 (35.23) | 158 (57.66) |  |
| Dyslipidemia (%) |  |  |  |  |  |  | <0.001 |
| No | 4640 (70.26) | 589 (96.08) | 701 (91.75) | 2470 (66.03) | 706 (58.25) | 174 (63.50) |  |
| Yes | 1964 (29.74) | 24 (3.92) | 63 (8.25) | 1271 (33.97) | 506 (41.75) | 100 (36.50) |  |

**Abbreviations**: CHNS, China Health and Nutrition Survey; CKM syndrome, Cardiovascular-Kidney-Metabolic syndrome; BMI, Body Mass Index; HbA1c, Hemoglobin A1c; HDL, High-Density Lipoprotein; LDL, Low-Density Lipoprotein; TC, Total Cholesterol; TG, Triglycerides; eGFR, estimated Glomerular Filtration Rate; SBP, Systolic Blood Pressure; DBP, Diastolic Blood Pressure.

**Table S4. Crude prevalence of Cardiovascular-Kidney-Metabolic Syndrome Stages Among Chinese Adults by Age and Sex in three national prospective cohorts.**

| Cohorts | N | Adults 30 years or older by cardiovascular-kidney-metabolic syndrome stage  (Prevalence, % (95% CI)) | | | | |
| --- | --- | --- | --- | --- | --- | --- |
|  |  | Stage 0 | Stage 1 | Stage 2 | Stage 3 | Stage 4 |
| **CHARLS** |  |  |  |  |  |  |
| 2011 Wave |  |  |  |  |  |  |
| Total | 9,077 | 7.5 (6.9-8.0) | 14.3 (13.6-15.1) | 40.8 (39.8-41.8) | 23.9 (23.0-24.8) | 13.5 (12.8-14.2) |
| Age |  |  |  |  |  |  |
| 45-64 y | 6,544 | 8.8 (8.1-9.5) | 17.3 (16.4-18.3) | 46.7 (45.5-47.9) | 15.7 (14.8-16.6) | 11.5 (10.8-12.3) |
| ≥ 65 y | 2,533 | 4.1 (3.4-4.9) | 6.6 (5.7-7.7) | 25.7 (24.0-27.4) | 45.1 (43.2-47.1) | 18.5 (17.0-20.0) |
| Sex |  |  |  |  |  |  |
| Female | 4,856 | 6.3 (5.7-7.0) | 16.8 (15.8-17.9) | 53.4 (52.0-54.8) | 8.6 (7.8-9.4) | 15.0 (14.0-16.0) |
| Male | 4,221 | 8.8 (8.0-9.7) | 11.5 (10.6-12.5) | 26.4 (25.1-27.8) | 41.6 (40.1-43.0) | 11.7 (10.8-12.7) |
| 2015 Wave |  |  |  |  |  |  |
| Total | 8,069 | 5.5 (5.0-6.0) | 19.3 (18.5-20.2) | 35.9 (34.8-36.9) | 26.6 (25.6-27.5) | 12.7 (12.0-13.5) |
| Age |  |  |  |  |  |  |
| 45-64 y | 4,880 | 7.3 (6.6-8.1) | 24.8 (23.6-26.0) | 41.6 (40.3-43.0) | 16.3 (15.3-17.4) | 9.9 (9.1-10.8) |
| ≥ 65 y | 3,189 | 2.7 (2.2-3.3) | 11.0 (10.0-12.1) | 27.1 (25.5-28.6) | 42.3 (40.6-44.0) | 17.0 (15.7-18.3) |
| Sex |  |  |  |  |  |  |
| Female | 4,386 | 5.0 (4.4-5.7) | 23.0 (21.8-24.3) | 49.0 (47.6-50.5) | 8.9 (8.1-9.8) | 14.1 (13.1-15.1) |
| Male | 3,683 | 6.1 (5.4-6.9) | 15.0 (13.8-16.1) | 20.2 (18.9-21.5) | 47.6 (46.0-49.2) | 11.1 (10.1-12.2) |
| **CLHLS** |  |  |  |  |  |  |
| 2012 Wave | 1,789 |  |  |  |  |  |
| Total |  | 2.9 (2.2-3.8) | 1.9 (1.4-2.6) | 22.3 (20.4-24.3) | 59.3 (57.0-61.6) | 13.6 (12.1-15.2) |
| Age |  |  |  |  |  |  |
| <80 y | 653 | 3.5 (2.4-5.2) | 3.2 (2.1-4.9) | 20.1 (17.2-23.3) | 59.7 (55.9-63.4) | 13.5 (11.1-16.3) |
| ≥ 80 y | 1136 | 2.6 (1.8-3.6) | 1.1 (0.7-2.0) | 23.6 (21.2-26.1) | 59.1 (56.2-61.9) | 13.6 (11.8-15.8) |
| Sex |  |  |  |  |  |  |
| Female | 936 | 4.4 (3.3-5.9) | 2.9 (2.0-4.2) | 41.1 (38.0-44.3) | 37.0 (33.9-40.1) | 14.6 (12.5-17.0) |
| Male | 853 | 1.3 (0.7-2.3) | 0.8 (0.4-1.7) | 1.6 (1.0-2.7) | 83.8 (81.2-86.1) | 12.4 (10.4-14.8) |
| 2014 Wave |  |  |  |  |  |  |
| Total | 1,035 | 4.9 (3.8-6.4) | 3.1 (2.2-4.3) | 18.2 (15.9-20.6) | 59.0 (56.0-62.0) | 14.8 (12.8-17.1) |
| Age |  |  |  |  |  |  |
| <80 y | 423 | 5.7 (3.7-8.3) | 3.8 (2.2-6.1) | 15.6 (12.3-19.4) | 58.4 (53.5-63.1) | 16.5 (13.1-20.4) |
| ≥ 80 y | 612 | 4.4 (2.9-6.4) | 2.6 (1.5-4.2) | 19.9 (16.8-23.3) | 59.5 (55.5-63.4) | 13.6 (10.9-16.5) |
| Sex |  |  |  |  |  |  |
| Female | 494 | 8.3 (6.0-11.1) | 5.3 (3.5-7.6) | 36.0 (31.8-40.4) | 35.2 (31.0-39.6) | 15.2 (12.1-18.7) |
| Male | 541 | 1.9 (0.9-3.4) | 1.1 (0.4-2.4) | 1.9 (0.9-3.4) | 80.8 (77.2-84.0) | 14.4 (11.6-17.7) |
| **CHNS** |  |  |  |  |  |  |
| 2009 Wave |  |  |  |  |  |  |
| Total | 6,604 | 9.3 (8.6-10.0) | 11.6 (10.8-12.4) | 56.6 (55.4-57.8) | 18.4 (17.4-19.3) | 4.2 (3.7-4.7) |
| Age |  |  |  |  |  |  |
| <45 y | 1,853 | 18.4 (16.7-20.2) | 17.9 (16.2-19.7) | 62.4 (60.2-64.6) | 1.0 (0.6-1.5) | 0.4 (0.2-0.8) |
| 45-64 y | 3,346 | 7.3 (6.5-8.3) | 11.4 (10.4-12.5) | 62.4 (60.8-64.1) | 15.6 (14.4-16.9) | 3.2 (2.7-3.9) |
| ≥ 65 y | 1,405 | 1.9 (1.3-2.8) | 3.8 (2.9-4.9) | 35.2 (32.8-37.8) | 47.8 (45.2-50.4) | 11.3 (9.8-13.1) |
| Sex |  |  |  |  |  |  |
| Female | 3,540 | 10.5 (9.5-11.6) | 14.4 (13.0-15.3) | 63.9 (62.3-65.4) | 7.5 (6.7-8.4) | 4.0 (3.4-4.7) |
| Male | 3,064 | 7.9 (7.0-8.9) | 8.7 (7.7-9.7) | 48.3 (46.5-50.0) | 30.9 (29.3-32.6) | 4.3 (3.6-5.1) |

**Abbreviations**: CHNS, China Health and Nutrition Survey; CHARLS, China Health and Retirement Longitudinal Study; CLHLS, Chinese Longitudinal Healthy Longevity.

**Table S5. Multivariable logistic regression for advanced CKM in three cohorts.**

| Variable | CHARLS 2011 | | CLHLS 2012 | | CHNS 2009 | |
| --- | --- | --- | --- | --- | --- | --- |
|  | OR (95%CI) | P | OR (95%CI) | P | OR (95%CI) | P |
| Age | 1.13 (1.12-1.14) | <0.001 | 1.14 (1.10-1.19) | <0.001 | 1.19 (1.18-1.21) | <0.001 |
| Sex |  |  |  |  |  |  |
| Female | Ref |  | Ref |  | Ref |  |
| Male | 2.22 (1.85-2.66) | <0.001 | 23.22 (11.72-46.00) | <0.001 | 5.02 (3.96-6.36) | <0.001 |
| Marriage |  |  |  |  |  |  |
| Married | Ref |  | Ref |  | Ref |  |
| Not married | 1.01 (0.83-1.24) | 0.889 | 1.35 (0.81-2.26) | 0.255 | 0.72 (0.56-0.91) | 0.006 |
| Education |  |  |  |  |  |  |
| >=primary high school | Ref |  | Ref |  | Ref |  |
| Illiterate | 0.74 (0.61-0.91) | 0.004 | 0.74 (0.32-1.70) | 0.481 | 0.77 (0.59-1.00) | 0.047 |
| Primary school | 1.08 (0.93-1.27) | 0.307 | 1.23 (0.57-2.67) | 0.596 | 1.02 (0.82-1.27) | 0.866 |
| Smoke |  |  |  |  |  |  |
| Non-smoking | Ref |  | Ref |  | Ref |  |
| Ever Smoking | 1.37 (1.09-1.71) | 0.006 | 0.81 (0.33-1.94) | 0.630 | 1.65 (1.13-2.42) | 0.010 |
| Smoking | 4.66 (3.83-5.67) | <0.001 | 3.77 (1.94-7.33) | <0.001 | 6.34 (5.06-7.94) | <0.001 |
| Drink |  |  |  |  |  |  |
| No | Ref |  | Ref |  | Ref |  |
| Yes | 0.80 (0.69-0.91) | 0.001 | 0.94 (0.50-1.77) | 0.859 | 0.68 (0.55-0.82) | <0.001 |
| BMI | 1.08 (1.02-1.15) | 0.008 | 1.13 (1.06-1.21) | <0.001 | 1.22 (1.19-1.25) | <0.001 |

**Abbreviations**: CHNS, China Health and Nutrition Survey; CHARLS, China Health and Retirement Longitudinal Study; CLHLS, Chinese Longitudinal Healthy Longevity; CKM syndrome, Cardiovascular-Kidney-Metabolic syndrome; BMI, Body Mass Index.

**Table S6. Age-by-sex interaction for advanced CKM in three cohorts.**

| Cohort | Interaction term | OR (95% CI) | P value |
| --- | --- | --- | --- |
| CHARLS 2011 | age × male | 1.078 (1.061-1.095) | <0.001 |
| CLHLS 2012 | age × male | 1.182 (1.054-1.326) | 0.004 |
| CHNS 2009 | age × male | 1.057 (1.039-1.076) | <0.001 |

**Abbreviations**: CHNS, China Health and Nutrition Survey; CHARLS, China Health and Retirement Longitudinal Study; CLHLS, Chinese Longitudinal Healthy Longevity.

**Table S7. Decomposition of Stage 3 CKM: contributions of CKD and CVD risk.**

| Sex | Contributions | CHARLS 2011 | | CLHLS 2012 | | CHNS 2009 | |
| --- | --- | --- | --- | --- | --- | --- | --- |
|  |  | Count | Percent, % | Count | Percent, % | Count | Percent, % |
| Female | Both (CVD risk + CKD) | 1 | 0.24 | 15 | 4.34 | 7 | 2.64 |
|  | CKD only | 2 | 0.48 | 17 | 4.91 | 5 | 1.89 |
|  | CVD risk only | 412 | 99.28 | 314 | 90.75 | 253 | 95.47 |
| Male | Both (CVD risk + CKD) | 12 | 0.68 | 36 | 5.03 | 5 | 0.53 |
|  | CKD only | 4 | 0.23 | 0 | 0.00 | 5 | 0.53 |
|  | CVD risk only | 1738 | 99.09 | 679 | 94.97 | 937 | 98.94 |

**Abbreviations**: CHNS, China Health and Nutrition Survey; CHARLS, China Health and Retirement Longitudinal Study; CLHLS, Chinese Longitudinal Healthy Longevity; CKM syndrome, Cardiovascular-Kidney-Metabolic syndrome; CKD, Chronic Kidney Disease; CVD, Cardiovascular Disease.

**Table S8. Comparison of baseline characteristics between included and excluded participants in the CHARLS 2011 cohort.**

| Variables | CHARLS 2011 | | | |
| --- | --- | --- | --- | --- |
|  | Excluded | Included | P | SMD |
| n | 8237 | 9077 |  |  |
| Age, years (mean (SD)) | 59.32 (10.48) | 59.53 (9.38) | 0.162 | 0.021 |
| Sex= Male (%) | 4200 (51.1) | 4221 (46.5) | <0.001 | 0.091 |
| Marriage = not married (%) | 1153 (14.0) | 1105 (12.2) | <0.001 | 0.055 |
| Education (%) |  |  | <0.001 | 0.189 |
| >=primary high school | 3127 (38.1) | 2652 (29.2) |  |  |
| Illiterate | 2068 (25.2) | 2666 (29.4) |  |  |
| primary school | 3012 (36.7) | 3758 (41.4) |  |  |
| Smoke (%) |  |  | <0.001 | 0.063 |
| Ex-smoker | 613 (8.2) | 797 (8.8) |  |  |
| Non-smoker | 4770 (63.9) | 5527 (60.9) |  |  |
| Smoker | 2078 (27.9) | 2753 (30.3) |  |  |
| Drink = Yes (%) | 2732 (33.7) | 2978 (32.8) | 0.213 | 0.019 |
| Height, cm (mean (SD)) | 158.38 (9.77) | 157.82 (9.07) | 0.001 | 0.059 |
| Weight, Kg (mean (SD)) | 58.97 (12.21) | 58.81 (11.63) | 0.463 | 0.013 |
| BMI (mean (SD)) | 24.56 (49.60) | 24.00 (27.71) | 0.407 | 0.014 |
| Creatinine, mg/dL (median [IQR]) | 0.77 [0.66, 0.90] | 0.76 [0.66, 0.88] | 0.004 | 0.049 |
| Glucose, mg/dL (median [IQR]) | 102.42 [93.96, 114.84] | 102.60 [94.50, 113.76] | 0.697 | 0.005 |
| HbA1c, % (median [IQR]) | 5.10 [4.80, 5.40] | 5.10 [4.90, 5.40] | <0.001 | 0.09 |
| HDL, mg/dL (median [IQR]) | 47.94 [38.27, 58.09] | 49.48 [40.59, 59.92] | <0.001 | 0.109 |
| LDL, mg/dL (median [IQR]) | 112.89 [91.24, 135.70] | 114.43 [93.17, 137.63] | 0.006 | 0.079 |
| TC, mg/dL (median [IQR]) | 188.27 [163.92, 215.34] | 190.59 [167.40, 215.34] | 0.02 | 0.045 |
| TG, mg/dL (median [IQR]) | 109.74 [78.76, 163.73] | 105.32 [75.22, 153.99] | 0.001 | 0.087 |
| eGFR, ml/min/1.72m^2^ (median [IQR]) | 83.64 [69.31, 97.64] | 86.00 [70.68, 98.03] | 0.009 | 0.059 |
| SBP, mmHg (mean (SD)) | 131.13 (21.65) | 130.66 (21.51) | 0.238 | 0.022 |
| DBP, mmHg (mean (SD)) | 76.32 (12.22) | 75.82 (12.15) | 0.025 | 0.041 |
| Diabetes = Yes (%) | 539 (19.8) | 759 (8.4) | <0.001 | 0.333 |
| Hypertension = Yes (%) | 2777 (51.7) | 3743 (41.2) | <0.001 | 0.211 |
| Dyslipidemia = Yes (%) | 611 (26.1) | 2238 (24.7) | 0.164 | 0.033 |

**Abbreviations**: CHARLS, China Health and Retirement Longitudinal Study; CKM syndrome, Cardiovascular-Kidney-Metabolic syndrome; BMI, Body Mass Index; HbA1c, Hemoglobin A1c; HDL, High-Density Lipoprotein; LDL, Low-Density Lipoprotein; TC, Total Cholesterol; TG, Triglycerides; eGFR, estimated Glomerular Filtration Rate; SBP, Systolic Blood Pressure; DBP, Diastolic Blood Pressure.

**Table S9. Comparison of baseline characteristics between included and excluded participants in the CLHLS 2012 cohort.**

| Variables | CLHLS 2012 | | | |
| --- | --- | --- | --- | --- |
|  | Excluded | Included | P | SMD |
| n | 183 | 1789 |  |  |
| Age, years (mean (SD)) | 87.77 (10.71) | 84.78 (11.91) | 0.001 | 0.264 |
| Sex = Male (%) | 82 (44.8) | 853 (47.7) | 0.507 | 0.058 |
| Marriage = not married (%) | 92 (64.3) | 1025 (57.6) | 0.135 | 0.139 |
| Education (%) |  |  | 0.48 | 0.097 |
| >=primary high school | 14 (7.7) | 179 (10.1) |  |  |
| Illiterate | 109 (60.2) | 1082 (61.0) |  |  |
| primary school | 58 (32.0) | 514 (29.0) |  |  |
| Smoke (%) |  |  | 0.647 | 0.087 |
| Ex-smoker | 9 (6.8) | 159 (8.9) |  |  |
| Non-smoker | 101 (76.5) | 1311 (73.3) |  |  |
| Smoker | 22 (16.7) | 319 (17.8) |  |  |
| Drink = Yes (%) | 16 (11.3) | 294 (16.5) | 0.131 | 0.151 |
| Height, cm (mean (SD)) | 154.62 (10.96) | 154.20 (11.05) | 0.618 | 0.039 |
| Weight, Kg (mean (SD)) | 50.96 (12.84) | 51.15 (13.20) | 0.853 | 0.015 |
| BMI (mean (SD)) | 21.20 (4.46) | 21.35 (4.32) | 0.636 | 0.036 |
| Creatinine, mg/dL (median [IQR]) | 0.93 [0.77, 1.11] | 0.87 [0.72, 1.03] | 0.011 | 0.134 |
| Glucose, mg/dL (median [IQR]) | 3.87 [2.80, 4.53] | 4.48 [3.76, 5.18] | <0.001 | 0.379 |
| HDL, mmol/l (median [IQR]) | 1.27 [1.05, 1.54] | 1.24 [1.03, 1.49] | 0.355 | 0.095 |
| LDL, mmol/l (median [IQR]) | 2.34 [1.79, 2.94] | 2.51 [1.99, 3.05] | 0.015 | 0.173 |
| TC, mmol/l (median [IQR]) | 4.14 [3.49, 4.84] | 4.24 [3.61, 4.92] | 0.284 | 0.081 |
| TG, mmol/l (median [IQR]) | 0.90 [0.66, 1.25] | 0.81 [0.59, 1.16] | 0.029 | 0.102 |
| eGFR, ml/min/1.72m^2^ (median [IQR]) | 56.45 [44.87, 69.54] | 61.20 [48.82, 74.51] | 0.001 | 0.249 |
| SBP, mmHg (mean (SD)) | 145.37 (25.08) | 140.19 (22.76) | 0.025 | 0.216 |
| DBP, mmHg (mean (SD)) | 77.55 (10.35) | 80.87 (12.00) | 0.006 | 0.296 |
| Diabetes = Yes (%) | 11 (7.1) | 156 (8.7) | 0.603 | 0.058 |
| Hypertension = Yes (%) | 90 (54.2) | 1072 (59.9) | 0.177 | 0.115 |
| Dyslipidemia = Yes (%) | 42 (23.0) | 421 (23.5) | 0.932 | 0.014 |

**Abbreviations**: CLHLS, Chinese Longitudinal Healthy Longevity; CKM syndrome, Cardiovascular-Kidney-Metabolic syndrome; BMI, Body Mass Index; HbA1c, Hemoglobin A1c; HDL, High-Density Lipoprotein; LDL, Low-Density Lipoprotein; TC, Total Cholesterol; TG, Triglycerides; eGFR, estimated Glomerular Filtration Rate; SBP, Systolic Blood Pressure; DBP, Diastolic Blood Pressure.

**Table S10. Comparison of baseline characteristics between included and excluded participants in the CHNS 2009 cohort.**

| Variables | CHNS 2009 | | | |
| --- | --- | --- | --- | --- |
|  | Excluded | Included | P | SMD |
| n | 1247 | 6604 |  |  |
| Age, years (mean (SD)) | 51.00 (12.49) | 53.51 (13.05) | <0.001 | 0.196 |
| Sex = male (%) | 648 (52.0) | 3540 (53.6) | 0.302 | 0.033 |
| Marriage = Not Married (%) | 158 (12.7) | 795 (12.0) | 0.562 | 0.019 |
| Education (%) |  |  | 0.016 | 0.088 |
| >=primary high school | 302 (24.2) | 1384 (21.0) |  |  |
| Illiterate | 298 (23.9) | 1758 (26.6) |  |  |
| primary school | 647 (51.9) | 3462 (52.4) |  |  |
| Smoke (%) |  |  | 0.504 | 0.035 |
| Ex-smoker | 50 (4.0) | 223 (3.4) |  |  |
| Non-smoker | 854 (68.5) | 4521 (68.5) |  |  |
| Smoker | 343 (27.5) | 1860 (28.2) |  |  |
| Drink = Yes (%) | 401 (32.2) | 2172 (32.9) | 0.637 | 0.016 |
| Height, cm (mean (SD)) | 162.88 (8.30) | 160.37 (8.48) | <0.001 | 0.299 |
| Weight, Kg (mean (SD)) | 62.88 (11.34) | 60.78 (11.22) | <0.001 | 0.186 |
| BMI (mean (SD)) | 23.61 (3.45) | 23.55 (3.45) | 0.579 | 0.018 |
| Creatinine, mg/dL (median [IQR]) | 0.94 [0.85, 1.05] | 0.96 [0.86, 1.10] | <0.001 | 0.089 |
| Glucose, mg/dL (median [IQR]) | 90.00 [82.71, 98.82] | 93.24 [85.86, 102.60] | <0.001 | 0.149 |
| HbA1c, % (median [IQR]) | 5.50 [5.30, 5.80] | 5.50 [5.20, 5.90] | 0.002 | 0.040 |
| HDL, mg/dL (median [IQR]) | 51.43 [43.70, 62.26] | 53.75 [45.24, 63.42] | <0.001 | 0.025 |
| LDL, mg/dL (median [IQR]) | 108.86 [88.07, 130.32] | 115.24 [93.19, 139.21] | <0.001 | 0.186 |
| TC, mg/dL (median [IQR]) | 179.81 [160.48, 208.04] | 187.55 [164.35, 213.84] | <0.001 | 0.146 |
| TG, mg/dL (median [IQR]) | 113.37 [76.62, 186.01] | 114.26 [77.95, 176.26] | 0.602 | 0.086 |
| eGFR, ml/min/1.72m^2^ (median [IQR]) | 80.78 [70.71, 91.07] | 76.78 [66.45, 87.22] | <0.001 | 0.263 |
| SBP, mmHg (mean (SD)) | 126.21 (17.65) | 126.34 (19.21) | 0.929 | 0.007 |
| DBP, mmHg (mean (SD)) | 81.87 (11.16) | 80.93 (11.18) | 0.247 | 0.085 |
| Diabetes = Yes (%) | 31 (2.5) | 220 (3.3) | 0.142 | 0.050 |
| Hypertension = Yes (%) | 163 (13.1) | 978 (14.8) | 0.12 | 0.050 |
| Dyslipidemia = Yes (%) | 347 (28.5) | 1964 (29.7) | 0.389 | 0.028 |

**Abbreviations**: CHNS, China Health and Nutrition Survey; CKM syndrome, Cardiovascular-Kidney-Metabolic syndrome; BMI, Body Mass Index; HbA1c, Hemoglobin A1c; HDL, High-Density Lipoprotein; LDL, Low-Density Lipoprotein; TC, Total Cholesterol; TG, Triglycerides; eGFR, estimated Glomerular Filtration Rate; SBP, Systolic Blood Pressure; DBP, Diastolic Blood Pressure.

**Table S11. Definition of CKM syndrome staging.**

| **CKM syndrome stages** | **Definition** |
| --- | --- |
| Stage 0: No CKM health risk factors | Individuals without overweight/obesity, metabolic risk factors (hypertriglyceridemia, hypertension, diabetes, MeTS), CKD or subclinical/clinical CVD  (1)BMI < 25 kg/m^2^  (2)WC <88/102 cm in female/male |
| Stage 1: Excess or dysfunctional adiposity | Individuals with overweight/obesity, abdominal obesity, or adipose tissue dysfunction without other metabolic risk factors, CKD, or subclinical/clinical CVD  (1)BMI ≥ 25 kg/m^2^  (2)WC ≥ 88/102 cm in female/male  (3)FBG levels ranging from 100 to 124 mg/dL, or HbA1c levels between 5.7 and 6.4 % |
| Stage 2: Metabolic risk factors and moderate to high-risk CKD | Individuals with metabolic risk factors (hypertriglyceridemia, hypertension, diabetes, MeTS*) or moderate to high-risk CKD stage  (1)TG >135 mg/dL  (2)Hypertension is defined by an SBP of ≥130 mm Hg, a DBP of ≥80 mm Hg, a medical diagnosis, or taking antihypertensive medication.  (3)Diabetes is defined by FBG levels of > 126 mg/dL, HbA1c levels of ≥ 6.5%, a medical diagnosis, or taking insulin or glucose-lowering medication.  (4) Moderate to high-risk CKD in the KDIGO classification is defined as UACR ≥ 30 mg/g and eGFR ≥ 60 ml/min/1.73m^2^, UACR < 300 mg/g and eGFR ≤ 45-59 ml/min/1.73m^2^, or UACR < 30 mg/g and eGFR ≤ 30-44 ml/min/1.73m^2^. |
| Stage 3: Subclinical CVD in CKM | Risk equivalents for subclinical CVD: high predicted 10-year CVD risk or very high-risk KDIGO CKD stage  (1)A high 10-year CVD risk is defined as a 20% or above risk.  (2)Very high-risk CKD in the KDIGO classification is defined as UACR ≥ 300 mg/g and eGFR ≤ 45-59 ml/min/1.73 m^2^, UACR ≥ 30 mg/g and eGFR ≤ 30-44 ml/min/1.73 m^2^, or eGFR ≤ 29 ml/min/1.73 m^2^. |
| Stage 4: Clinical CVD in CKM | Clinical CVD (self-reported diagnosed cardiovascular disease, including heart failure, coronary heart disease, angina, heart attack, and stroke) in individuals |
| **Abbreviations**: CKM syndrome, Cardiovascular-Kidney-Metabolic syndrome; CKD, chronic kidney disease; CVD, cardiovascular disease; BMI, body mass index; WC, waist circumference; FBG, fasting blood glucose; HbA1c, glycated hemoglobin A1c; KDIGO, Kidney Disease Improving Global Outcomes; UACR, urine albumin-to-creatinine ratio; eGFR, estimated glomerular filtration rate; TG, triglycerides; SBP, systolic blood pressure; DBP, diastolic blood pressure. | |
| * MeTS is defined by the presence of ≥3 of the following: (1) WC ≥ 102 cm for men or ≥ 88 cm for women; (2) HDL-C <40 mg/dL for men, <50 mg/dL for women; (3) TG ≥150 mg/dL; (4) Elevated blood pressure (SBP ≥130 mm Hg, DBP ≥80 mm Hg, a medical diagnosis, or taking antihypertensive medication); (5) FBG ≥100 mg/dL | |

**Figure S1. Prevalence and trends by gender in cardio-kidney-metabolic syndrome stages 1-2 vs. 3-4.**

(**A**) Longitudinal changes in the prevalence of early- and advanced-stage CKM in the CHARLS database, stratified by gender. (**B**) Longitudinal changes in the prevalence of early- and advanced-stage CKM in the CLHLS database, stratified by gender. (C) Prevalence of early- and advanced-stage CKM in the CHNS database, stratified by gender.

The x-axis represents the different waves of the three cohorts, and the y-axis denotes the prevalence of different CKM stages among the population in a specific wave of each database. Black dots represent the prevalence rates for stage 1-2, and gray dots represent stage 3-4. The prevalence rates for each time point are depicted as dots. The segmented lines connecting the dots illustrate the changing trends of prevalence by early or advanced CKM stages over the study period.

**Abbreviations**: CHNS, China Health and Nutrition Survey; CHARLS, China Health and Retirement Longitudinal Study; CLHLS, Chinese Longitudinal Healthy Longevity; CKM, Cardiovascular-Kidney-Metabolic.

**STROBE Statement**—checklist of items that should be included in reports of observational studies

|  | | Item No | Recommendation |
| --- | --- | --- | --- |
| **Title and Abstract** | | 1 | (*a*) Indicate the study’s design with a commonly used term in the title or the Abstract  **Included (Abstract)** |
|  |  |  | (*b*) Provide in the Abstract an informative and balanced summary of what was done and what was found  **Included (the 2-4 sentence of Abstract)** |
| Introduction | | | |
| Background/rationale | | 2 | Explain the scientific background and rationale for the investigation being reported  **Included (page 4, lines 43-52)** |
| Objectives | | 3 | State specific objectives, including any prespecified hypotheses  **Included (page 4, lines 54-58)** |
| Methods | | | |
| Study design | | 4 | Present key elements of study design early in the paper  **Included (pages 9-10, lines 174-176)** |
| Setting | | 5 | Describe the setting, locations, and relevant dates, including periods of recruitment, exposure, follow-up, and data collection  **Included (page 10, lines 176-179)** |
| Participants | | 6 | (*a*) *Cohort study*—Give the eligibility criteria, and the sources and methods of selection of participants. Describe methods of follow-up  **Included (page 10, lines 179-180)**  *Case-control study*—Give the eligibility criteria, and the sources and methods of case ascertainment and control selection. Give the rationale for the choice of cases and controls  *Cross-sectional study*—Give the eligibility criteria, and the sources and methods of selection of participants |
|  |  |  | (*b*) *Cohort study*—For matched studies, give matching criteria and number of exposed and unexposed  *Case-control study*—For matched studies, give matching criteria and the number of controls per case |
| Variables | | 7 | Clearly define all outcomes, exposures, predictors, potential confounders, and effect modifiers. Give diagnostic criteria, if applicable  **Included (page 10, lines 183-189)** |
| Data sources/ measurement | | 8* | For each variable of interest, give sources of data and details of methods of assessment (measurement). Describe comparability of assessment methods if there is more than one group  **Included (pages 9-10, lines 174-176)** |
| Bias | | 9 | Describe any efforts to address potential sources of bias  **Included (page 9, lines 162-163)** |
| Study size | | 10 | Explain how the study size was arrived at  **Included (page 4, lines 59-63)** |
| Quantitative variables | | 11 | Explain how quantitative variables were handled in the analyses. If applicable, describe which groupings were chosen and why  **Included (Not** applicable**)** |
| Statistical methods | | 12 | (*a*) Describe all statistical methods, including those used to control for confounding  **Included (Statistical analysis in Methods)** |
|  |  |  | (*b*) Describe any methods used to examine subgroups and interactions  **Included (page 11, line 199)** |
|  |  |  | (*c*) Explain how missing data were addressed  **Included (page 11, lines 201-202)** |
|  |  |  | (*d*) *Cohort study*—If applicable, explain how loss to follow-up was addressed  *Case-control study*—If applicable, explain how matching of cases and controls was addressed  *Cross-sectional study*—If applicable, describe analytical methods taking account of sampling strategy  **Included (Not** applicable**)** |
|  |  |  | (*e*) Describe any sensitivity analyses  **Included (page 11, lines 201-202)** |
| Results | | | |
| Participants | 13* | (a) Report numbers of individuals at each stage of study—eg numbers potentially eligible, examined for eligibility, confirmed eligible, included in the study, completing follow-up, and analysed  **Included (pages 4-5, lines 59-65)** | |
|  |  | (b) Give reasons for non-participation at each stage  **Included (Not** applicable**)** | |
|  |  | (c) Consider use of a flow diagram  **Included ( (Not** applicable**))** | |
| Descriptive data | 14* | (a) Give characteristics of study participants (eg demographic, clinical, social) and information on exposures and potential confounders  **Included (Table 1)** | |
|  |  | (b) Indicate number of participants with missing data for each variable of interest  **Included (Table 1)** | |
|  |  | (c) *Cohort study*—Summarise follow-up time (eg, average and total amount)  **Included (Figure 1)** | |
| Outcome data | 15* | *Cohort study*—Report numbers of outcome events or summary measures over time  **Included (Table 1 and 2, Figure 1)** | |
|  |  | *Case-control study—*Report numbers in each exposure category, or summary measures of exposure | |
|  |  | *Cross-sectional study—*Report numbers of outcome events or summary measures | |
| Main results | 16 | (*a*) Give unadjusted estimates and, if applicable, confounder-adjusted estimates and their precision (eg, 95% confidence interval). Make clear which confounders were adjusted for and why they were included  **Included (Table 2, Figure 2)** | |
|  |  | (*b*) Report category boundaries when continuous variables were categorized | |
|  |  | (*c*) If relevant, consider translating estimates of relative risk into absolute risk for a meaningful time period | |
| Other analyses | 17 | Report other analyses done—eg analyses of subgroups and interactions, and sensitivity analyses  **Included (Table 1-11, Figure S1)** | |
| Discussion | | | |
| Key results | 18 | Summarise key results with reference to study objectives  **Included (page 7, lines 109-120)** | |
| Limitations | 19 | Discuss limitations of the study, taking into account sources of potential bias or imprecision. Discuss both direction and magnitude of any potential bias  **Included (page 9, 162-166)** | |
| Interpretation | 20 | Give a cautious overall interpretation of results considering objectives, limitations, multiplicity of analyses, results from similar studies, and other relevant evidence  **Included (page 9, 162-166)** | |
| Generalisability | 21 | Discuss the generalisability (external validity) of the study results  **Included (page 9, 167-170)** | |
| Other information | | | |
| Funding | 22 | Give the source of funding and the role of the funders for the present study and, if applicable, for the original study on which the present article is based  **Included (Acknowledgements)** | |

*Give information separately for cases and controls in case-control studies and, if applicable, for exposed and unexposed groups in cohort and cross-sectional studies.

**Note:** An Explanation and Elaboration article discusses each checklist item and gives methodological background and published examples of transparent reporting. The STROBE checklist is best used in conjunction with this article (freely available on the Web sites of PLoS Medicine at http://www.plosmedicine.org/, Annals of Internal Medicine at http://www.annals.org/, and Epidemiology at http://www.epidem.com/). Information on the STROBE Initiative is available at www.strobe-statement.org.
